# Understanding ethics of reporting health related events in media: A qualitative phenomenological study in Karachi and Peshawar

**DOI:** 10.12669/pjms.40.3.7414

**Published:** 2024

**Authors:** Shiraz Shaikh, Aroosa Nadeem, Mehjabeen Musharraf, Sheraz Fazid

**Affiliations:** 1 Shiraz Shaikh, FCPS. Associate Professor, APPNA Institute of Public Health, Jinnah Sindh Medical University, Karachi, Pakistan; 2 Aroosa Nadeem, MBBS, MSPH. Research Associate, APPNA Institute of Public Health, Jinnah Sindh Medical University, Karachi, Pakistan; 3 Mehjabeen Musharraf, M.Sc, MSPH. Senior Lecturer, APPNA Institute of Public Health, Jinnah Sindh Medical University, Karachi, Pakistan; 4 Sheraz Fazid, MPH. Epidemiologist, Institute of Public Health & Social Sciences, Khyber Medical University, Peshawar, Pakistan

**Keywords:** Media, Health reporting, Ethics, coverage of health events, Media reporting ethics

## Abstract

**Objective:**

The specific objectives of this study were to identify the ethical issues in reporting of health-related events in media and suggest ways to improve it.

**Method:**

This was a qualitative phenomenological study conducted by APPNA Institute of Public Health, Jinnah Sindh Medical University in collaboration with Institute of Public Health, Khyber Medical University from January to April 2022. A total of 38 focus group discussions and in-depth interviews were conducted either face to face at place of convenience of interviewees or online. The participants were health reporters, healthcare workers (HCWs), and representatives of law enforcement agencies in two cities i.e., Karachi and Peshawar. Data were analyzed by using the deductive and inductive approaches, by four independent experts including the Principal Investigator (PI) and three research fellows.

**Results:**

Ethical issues related to health reporting in the field included interference of the reporters in rescue efforts during an emergency and interference in emergency medical care of the victims. In reporting, careless disregard for patient confidentiality and privacy; using unreliable sources of information; using wrong terminology; sensationalizing the news and jumping to conclusions in cases of malpractice were reported as main problems. Negative influences on health reporting included poor training of the reporters on health reporting ethics, organizational pressures, and lack of cooperation by relevant health authorities.

**Conclusion:**

The quality of health reporting can be improved by building the capacity of health reporters in understanding the ethical issues and their social responsibilities toward health.

## INTRODUCTION

Media organizations play an important role in disseminating news regarding healthcare events which assists in improving response to any medical crisis.^1^-^3^ They also help in educating people on how to avoid medical emergencies and dispel myths related to different diseases.^3^,^4^ Over the last couple of decades, numerous organizations in print and electronic media have mushroomed and competition for ratings has increased between them.^4^ This has led to careless reporting of deaths and serious cases, particularly in mass casualties.^5^ In medical emergencies and disasters, a lack of coordinated response from healthcare, rescue, and media organizations has been observed. This has resulted in the development of stressful situations which are the main triggers of violence against healthcare workers.^6^,^7^

The quality of reporting has come under serious scrutiny and general ethical codes have been developed.^8^-^10^ However, specific issues that arise in reporting different scenarios of healthcare have not been broadly covered.^3^,^11^ There is a serious need to assess the relevant ethical standards being taught and implemented, and the different factors influencing the reporting behavior of the health reporters.^3^,^6^,^7^ This study aimed to identify the ethical issues in reporting of health related events in media and suggest ways to improve it.

## METHODS

This was a qualitative transcendental phenomenological study conducted by APPNA Institute of Public Health, Jinnah Sindh Medical University in collaboration with Khyber Medical University in Karachi and Peshawar from January to April 2022.

Consolidated criteria for reporting qualitative Research (COREQ guidelines) have been followed for the planning, execution, and writing of this study.^12^ The validity and reliability of this qualitative study were taken care of by adopting Guba’s constructs of trustworthiness.^13^ Before starting data collection, the first two authors penned down their views. According to the PI, health reporting is usually not scientific which doesn’t help create health awareness and mitigate myths and misconceptions. The opinion of the second author about the topic was that media has an important role in shaping public opinion about health care workers. Therefore, journalists in Pakistan must be properly trained in reporting emergencies and disasters.

All researchers were public health practitioners and trained in qualitative data analysis. Focus Group Discussions (FGDs) and In-depth interviews (IDIs) were conducted at a place of convenience or online. Representatives of media, healthcare professionals and law enforcing agencies were purposively sampled. Media representatives included health reporters, chief editors, bureau chiefs and journalism academics. Medical officers, nursing staff, departmental in-charges and media managers were interviewed from hospitals. Representatives of LEAs comprised of policemen and security guards and managers.

All FGDs and IDIs were conducted by two trained research fellows. In each FGD 4-8 participants were interviewed. The details of the participants are provided in Table1a and Table 1b. The individual and institutional identities of all participants have been kept confidential on their request.

**Table-I(a):** List of FGDs and their participants.

FGD No:	City	Participants
1	Karachi	Four Print media Health Reporters
2	Peshawar	Six Print media Health Reporters
3	Peshawar	Four Print media Health Reporters
4	Karachi	Four Print and Electronic Media Health Reporters
5	Karachi	Four Electronic Media Health Reporters
6	Peshawar	Eight Electronic Media Health Reporters
7	Peshawar	Five Electronic Media Health Reporters
8	Peshawar	Five Electronic Media Health Reporters
9	Peshawar	Four Electronic Media Health Reporters
19	Karachi	Four Regional Language Health Reporters
11	Karachi	Four Regional Language Health Reporters
12	Karachi	Four policemen
13	Peshawar	Four Security Guards of a public sector hospital
13	Karachi	Six Healthcare Workers of Emergency Department of public sector hospital
14	Peshawar	Six Healthcare Workers of Emergency Department of public sector hospital
15	Peshawar	Six Ambulance Drivers

**Table-I(b):** List of IDIs and their participants.

IDI No:	City	Participants
1	Karachi	Editor in Chief of an Electronic Media Organization
2	Peshawar	Bureau Chief of an Electronic Media Organization
3	Peshawar	Bureau Chief of an Electronic Media Organization
4	Peshawar	Bureau Chief of an Electronic Media Organization
5	Karachi	Editor in Chief of a Print Media Organization
6	Peshawar	Editor in Chief of a Print Media Organization
7	Karachi	Managing Director of a media organization
8	Karachi	Renowned Journalist with background on work on Journalism Ethics
9	Karachi	Faculty Mass Communication in a public sector University
10	Karachi	Founding Director of a Private Sector Academic Institution
11	Karachi	Medical Officer in Emergency of a public sector tertiary care hospital
12	Karachi	In charge Emergency of a public sector tertiary care hospital
13	Karachi	In charge of a Trauma care center of public sector tertiary care hospital
14	Peshawar	Media Manager of public sector tertiary care hospital
15	Peshawar	Media Manager of public sector tertiary care hospital
16	Peshawar	Media Manager of private sector tertiary care hospital
17	Peshawar	Media Manager of private sector tertiary care hospital
18	Karachi	Head of Clinical Training in an Ambulance Organization
19	Karachi	Media Manager in an Ambulance Organization
20	Karachi	SHO Karachi Police

They were audio-taped along-with notes taken by a research assistant. The average duration of the interviews was 30-45 minutes. To maintain credibility, the participants were encouraged to talk about the details so that the phenomenon unfolds with all its completeness and comprehensiveness. The responses of the participants were not influenced by interviewers and the questions were open-ended. All participants were assured of anonymity and confidentiality of their personal and occupational identities.

With the journalists FGDs and IDIs focused on their views on ethics on health reporting taught during their training, ethical dilemmas they face about what and how to report and barriers and challenges that they face in ethically reporting healthcare events. With other stakeholders interviews focused on their positive and negative experiences with health reporters, situations which could have been handled differently by health reporters according to their opinion and areas which they felt the journalists needed to work on to improve their reporting

Manual content analysis was done. Transcription of the FGDs and IDIs was done using the audio-video recording and double-checked with the notes taken by the note taker. To ensure dependability, random audio and video records were reviewed for accuracy. After doing three interviews, inductive analysis was done, and themes were generated. This was followed by the deductive analysis of all transcripts. Deductive analysis formulated the sub-themes by merging the repetitive ideas, this was a thorough and highly iterative process. Themes and subthemes were developed and all sentences matching the themes and subthemes were fitted into the analysis. The analysis was done by four experts (PI and three research fellows) separately and then the researchers met to finalize the results with consensus.

### Ethical considerations

Permission to carry out the research was obtained from the ‘Independent review board’ (IRB) of Jinnah Sindh Medical University, Karachi (Approval number JSMU/IRB/2021/-559, dated October 7, 2021) and Khyber Medical University, Peshawar (Approval number DIR/KMU-EB/DP/00083, dated September 16, 2021) respectively. Informed consent was verbally sought from the participants (mostly professionals) after explaining to them the relevant details of the project, before proceeding with the interviews.

## RESULTS

Overall, 38 FGDs and IDIs were conducted with electronic and print media journalists (12), healthcare workers and administrators (12), journalism academia (10), and representatives of law enforcement agencies (4) in Karachi and Peshawar. From the transcripts, the following themes and sub-themes were identified.

### Ethical Issues and Dilemmas in Health reporting:

### Interference in the rescue of victims in the field during an emergency

The ambulance workers and policemen complained that media workers interfered in the rescue operations by not keeping a safe distance and trying to talk to injured victims. A police officer said, “Media interferes with our work just for sake of breaking the news and ratings, they break the cordoned-off area and put their mike on our face.” However, the journalists defended themselves by claiming their right to information. A chief editor said, “For journalists: the right to know, right to information and right to expression are basic human rights.”

### Interference in care of patients receiving medical care in an emergency

Healthcare workers also complained of journalists not being respectful to hospital protocols and disrupting process of care. A doctor said, “Unfortunately, they even stand by the bed site when we are giving emergency care, but in our set up you can’t ask anyone to leave.” Conversely, journalists complained that hospitals did not explain the hospital protocols to them satisfactorily.

### Careless disregard for patient privacy and confidentiality

Healthcare workers believed that the patients’ rights of confidentiality and privacy were violated by health reporters, especially when reporting about famous personalities. Journalists argued that patient’s identities needed to be revealed for providing support to the disaster victims. A health reporter said, “Help will only come if details of the individual suffering are shown, do we need permission to do an act of kindness.” During emergencies, the media is responsible to provide information about the casualties. As expressed by a chief editor, “In disastrous situations like a plane crash, names are important because people want to know if their loved ones are victims or not”. Regarding public figures, a senior journalist commented, “I understand privacy, but some people are larger than life, therefore it is all right to take their pictures in hospitals.”

### Use of unreliable source of information

Some HCWs blamed the health reporters for publishing information regarding their facilities without any verification. An administrator of a trauma center said, “Media reporters get faulty information from guards and ward boys and base their exaggerated reports on that.” However, journalists also felt that the hospital authorities don’t permit highlighting deficiencies in the healthcare system due to a lack of mutual trust and to safeguard their own interests. A journalist said, “The hospital management tries to hide facts from the reporters, when facts are hidden, ethics are bypassed.”

### Use of wrong terminology/Misreporting

Healthcare workers also thought that the quality of health reports published in the media was limited by their poor understanding of the health system and medical terminologies. A junior doctor told, “Unfortunately media people try to influence our decisions although they have absolutely no knowledge on who should get admitted and who should not.” They also thought that due to their limited understanding of the process of care they found it difficult to differentiate between negligence and a natural consequence. A doctor expressed, “If a patient has a severe head injury and dies, his death cannot be attributed to negligence.”

The journalists also agreed that poor understanding of health terminology and care processes affected the quality of reporting, “Most of the health reporters don’t know what a pathogen is, how it gets transmitted and still they write stories on infectious diseases” Another journalist said, “They don’t understand how medicines work and what are their risks.”

### Sensationalism in the news

The dramatization of real-life suffering should be avoided. The tone and words in which any health event is reported must show empathy and realization that this sensationalism will affect the mental health of the families of victims.

### Jumping to conclusions in cases of malpractice

Some healthcare workers complained that journalists do not understand the holistic picture and blame healthcare workers for the lack of facilities. Director of emergency in a public sector hospital expressed, “We cannot run things the way we want them to because of lack of resources, and media reporters need to understand that”.

### Negative influences on reporting:

### Academic

Lack of relevant content in the curriculum was expressed as the main reasons for bad reporting on health. One of the journalists commented, “We are taught about crime reporting and political reporting, but health reporting is not taught at all.” Another journalist pointed out to lack of a specific code of health reporting ethics, “There is a code of ethical reporting but there is nothing specific to health reporting”.

### Organizational

The three most cited organizational reasons included low priority of the media organization to develop capacity in health reporting, hiring of unqualified reporters with limited understanding of health issues, and pressure of ratings to produce news in haste. Elaborating on these organizational barriers, a reporter complained, “The management discourages us to go for any training, if we request for attending a three day training, we are asked to resign first.” Another correspondent of print media said, “There are many journalists reporting healthcare who even do not have a qualification in journalism.” Another field reporter said, “There is a lot of pressure on us to report the details of the incident as quickly as possible, every channel wants to be the first to report any event”.

Less commonly reported reasons included high workload on journalists leading to low focus on health news, interest in producing sensation, and pressures of sponsors e.g., pharmaceuticals and powerful groups e.g., political parties The Chief Editor of a news agency commented, “A news agency may not publish the story of a drug reaction because the manufacturing pharmaceutical provides ads to the paper”.

### Health System Related Reasons

The most common reason for the poor health reporting quality was stated as the lack of proper mechanism in hospitals and health offices information-sharing with journalists. A senior journalist commented, “Media needs a source of authenticity, when that source does not exist or is not responsive, then they try to get information from here and there and air it on the basis of speculation”.

A few of the healthcare workers also confirmed non-availability of trained focal persons and clear protocols for sharing information with the media. A hospital administrator said, “To be honest, the media cell of the health department is very weak, there is no central point from where information can be obtained”.

### External Reasons

Government influence on media organizations to hide their deficiencies was also reported as a barrier to quality health reporting. Poor regulation by PEMRA was quoted as another reason, as pointed out by one of the journalists, “PEMRA takes notice mostly on political issues rather than ethical issues.”

## DISCUSSION

This is the first study in Pakistan to highlight problems with the media in reporting healthcare events and develop relevant ethical standards. Implementation of these guidelines will help improve coordination between the healthcare organizations and the media, prevent misinformation leading to panic amongst the public, while at the same time protecting the patients’ rights of privacy and dignity.

Health reporting has been found to be low on the priority of publishers, therefore, their commitment to building capacity in this area also has been limited.^2^,^14^,^15^ The journalists lack the necessary specialized training when dealing with the intricacies of health news.^2^,^15^ As a result, their capacity to report health news about different diseases has low precision and high sensation.^16^ Health reporters are also stated to put less effort into gathering authentic information from medical personnel and their ability to understand health research and terminology is also limited.^17^,^18^ Their deficiency in reporting authentic health news is also compounded by a lack of cooperation from the health experts in providing access to information.^3^,^19^,^20^ As a result, there is a situation of distrust between healthcare workers and health reporters leading to misinformation for the public.^21^ The quality of health reporting can only be improved by building the capacity of health reporters in understanding the ethical issues of health reporting.^15^

A training is proposed to be developed based on the issues identified in this study. First, they need to be oriented with the rights of HCWs and patients so that issues of confidentiality and privacy can be addressed. Second, they need to be explained the protocols of reporting emergencies in the field and in hospitals. Third, sources of authentic information shall be shared with them, which may include health experts, health officials, research journals, or websites for proper referencing of the shared information. Fourth, they shall be made to realize their social responsibility towards people in seeking health related information and in dispelling the circulating myths. Fifth, they should not try to sensationalize health news which may create unnecessary panic among the audience. Last, health reporters should understand that mishaps in healthcare seldom happen due to the negligence of healthcare workers and more often due to a lack of resources. They should work closely with health authorities to highlight systemic issues.

To address the academic, organizational, and social barriers, the training should be part of the routine curriculum for graduating students and on-job training for employees reporting health. After the training, regulatory authorities shall monitor the progress made in the quality of reporting.

## CONCLUSION

The quality of health reporting can be improved by building the capacity of health reporters in understanding the ethical issues and their social responsibilities toward health. Media organizations should make sure that none of the violations of ethical reporting happen and the quality of health reporting should be improved by building the capacity of health reporters in understanding the rights of HCW s and patients, protocols of reporting emergencies, reliable sources of information and their social responsibilities towards health. Moreover, all hospitals should establish a media cell and develop coordination with health reporters to ensure the smooth sharing of information.

### Authors’ Contribution:

**SS:** Conceived the idea, developed the proposal, trained data collectors, performed analysis, and prepared the first draft of the manuscript. Responsible and accountable for the accuracy or integrity of the work.

**AN:** Did literature search, conducted in-depth interviews and focus group discussions, performed analysis, and reviewed the manuscript.

**MM:** Did literature search, analysis, and manuscript writing.

**SF:** Conducted in-depth interviews and focus group discussions in Peshawar, performed analysis and reviewed the manuscript.
